# Comparative Transcriptome of Wild Type and Selected Strains of the Microalgae *Tisochrysis lutea* Provides Insights into the Genetic Basis, Lipid Metabolism and the Life Cycle

**DOI:** 10.1371/journal.pone.0086889

**Published:** 2014-01-29

**Authors:** Gregory Carrier, Matthieu Garnier, Loïc Le Cunff, Gaël Bougaran, Ian Probert, Colomban De Vargas, Erwan Corre, Jean-Paul Cadoret, Bruno Saint-Jean

**Affiliations:** 1 IFREMER-PBA, Nantes, France; 2 CNRS-UPMC, UMR 7144, Station Biologique de Roscoff, Roscoff, France; 3 UMT Geno-Vigne®, Montpellier, France; 4 CNRS-UPMC, ABiMS, Station Biologique de Roscoff, Roscoff, France; University of Granada - Q1818002F, Spain

## Abstract

The applied exploitation of microalgae cultures has to date almost exclusively involved the use of wild type strains, deposited over decades in dedicated culture collections. Concomitantly, the concept of improving algae with selection programs for particular specific purposes is slowly emerging. Studying since a decade an economically and ecologically important haptophyte *Tisochrysis lutea* (Tiso), we took advantage of the availability of wild type (Tiso-Wt) and selected (Tiso-S2M2) strains to conduct a molecular variations study. This endeavour presented substantial challenges: the genome assembly was not yet available, the life cycle unknown and genetic diversity of Tiso-Wt poorly documented. This study brings the first molecular data in order to set up a selection strategy for that microalgae.

Following high-throughput Illumina sequencing, transcriptomes of Tiso-Wt and Tiso-S2M2 were *de novo* assembled and annotated. Genetic diversity between both strains was analyzed and revealed a clear conservation, while a comparison of transcriptomes allowed identification of polymorphisms resulting from the selection program. Of 34,374 transcripts, 291 were differentially expressed and 165 contained positional polymorphisms (SNP, Indel). We focused on lipid over-accumulation of the Tiso-S2M2 strain and 8 candidate genes were identified by combining analysis of positional polymorphism, differential expression levels, selection signature and by study of putative gene function. Moreover, genetic analysis also suggests the existence of a sexual cycle and genetic recombination in *Tisochrysis lutea*.

## Introduction

Interest in microalgae as a potential source of economic benefit is booming [1]. Applications are envisaged in very different domains such as human or animal alimentation due to their high nutritional value [2], bio-remediation such as water purification [3], and pigment production for the cosmetic industry, for food-processing or for human health [4]. In the last decade new biotechnological applications have emerged [5]. The use of microalgae as cell factories would offer numerous advantages for the production of safe and complex recombinant pharmaceutical proteins [6]–[8]. Recently, several research projects have focused on the use of microalgae for biofuel production [9], citing very attractive biomass and oil productivities compared to oleaginous land plants. Mass production of microalgae would not enter into competition with agricultural food production as they can be cultivated on non-arable land, saline or wastewater sources [10]. In the context of the energy crisis, certain concepts of production of biofuels from algal oils are currently at the demonstration stage. However, the production of microalgae is currently not sufficiently economically efficient compared to the use of fossil fuels to be envisioned at a large scale [7].

Among the very large diversity of microalgae, a culture strain originally isolated from Tahiti and designated as *Isochrysis* affinis *galbana* but recently renamed *Tisochrysis lutea* (Tiso) [11] has been historically extensively studied due to it's widespread use in aquaculture as a feedstock for shellfish and shrimps that reflects an attractive fatty acid content [12]. However, the lack of molecular data for this strain has limited investigation of aspects such as the metabolism and life cycle of this microalga. Rapid developments in Next Generation Sequencing (NGS) technologies now make it relatively easy to conduct large-scale genotyping of species for which full genome sequences are not yet available [13].

Beyond the obvious economic interest of Tiso, it is also of significant fundamental interest as a member of a microalgal lineage (the division Haptophyta) which is diverse and often ecologically dominant in the planktonic photic realm [14]. Tiso is a member of the haptophyte order Isochrysidales that comprises two families, the Isochrysidaceae and the Noëlaerhadaceae. Members of the Noëlaerhabdaceae exhibit a heteromorphic haplo-diploid life cycle in which the diploid phase produces calcified plates (coccoliths) and the haploid phase is non-calcifying [14]. The best-known member of this family is *Emiliania huxleyi*, which is by far the most abundant coccolithophore in modern oceans and consequently an extremely important actor in global carbon cycling. By contrast, isochrysidaceaens such as Tiso have only one known non-calcifying morphological form. No information about sexual reproduction or ploidy levels are available for this family [16], [17].

Sophisticated selection programs like those implemented in terrestrial agriculture will probably play an important role in the future exploitation of microalgal resources [18]. Domestication of plants and animals by long-term selection programs led to the rise of modern agriculture. For example, the yield of wheat culture has increased 16-fold in 1200 years through directed selection of highly productive strains [11]. By comparison, domestication of microalgae is in its infancy. Recent studies in this domain have focused, for example, on production of the pigment prodigiosin in the rhodophyte *Hahella chejuensis* [19], carotenoid production in *Dunaliella salina* [20], and lipid production in *Nannochloropsis* sp. [21] and in Tiso [22]. Until recently, all these selection programs of microalgae consisted to identify and select the best individuals among a population. To date, microalgal selection programs generally start without prior knowledge of the natural diversity of the taxon in question. A number of studies have reported high levels of intra-specific genetic or metabolic diversity in different microalgal species [23]–[25]. In this study, we estimated the genetic diversity in a Tiso culture strain, this being a requirement for conception of improved selection programs [18]. Life cycles of microalgae are generally poorly known and consequently breeding programs have yet to be initiated, despite several real advances in the knowledge of certain microalgal groups such as diatoms [18].

In our laboratory, a selection program was performed starting from a wild type strain of *Tisochrysis lutea* (Tiso-Wt). This wild type strain consists in one ecotype (Tahiti) and is presumed to be composed of several genotypes characterizing a population of Tiso with an unknown diversity. A sequential mutation-selection procedure was performed from this wild type strain, involving: i) UVc treatment to induce mutations and thus increase the genetic (and metabolic) diversity of the strain, and ii) selection of the 10% of cells with the highest lipid content. This resulted in selection of a new certificate strain [26] (Tiso-S2M2) that accumulates twice the amount of neutral lipids in nitrogen limited culture conditions compared to the Tiso-Wt strain [22].

In context of algae selection program, this study using RNAseq approach, brings the first molecular data for an economically and ecologically important microalgae *Tisochrysis lutea*. Here, we describe the impact of a selection program on the genetic repertoire and gene expression of a selected strain. Comparison of transcriptomes of wild type and selected strains allowed identification of positional polymorphism and differentially expression levels. Analysis of polymorphisms generated as a result of the selection program provides detailed information on the genomic-level impact of the program, and notably identification of candidate genes that could account for lipid over-accumulation, as well as providing insights into the putative life cycle of this species.

## Results

### *De novo* sequencing and annotation of Tiso transcriptomes

**Figure 1:**
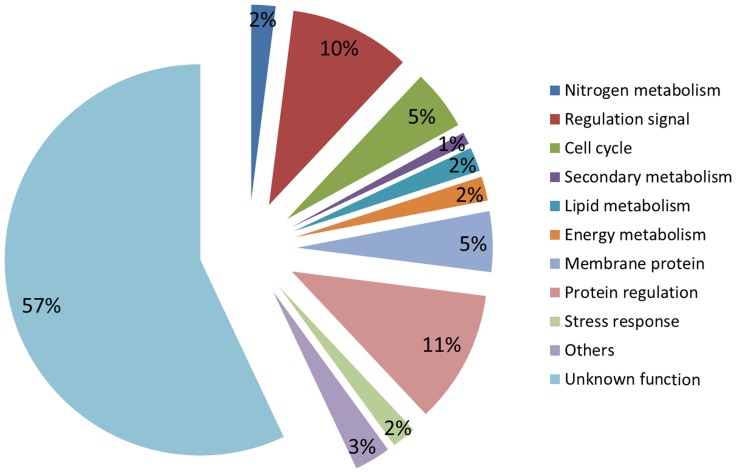
Annotation of Tiso transcriptome. Transcripts were annotated by BLAST in the NCBI nr database. Transcripts were sorted among identified major cell functions from Gene Ontology.

Transcriptomes of two Tiso strains, the reference strain (Tiso-Wt) and a selected strain (Tiso-S2M2), were sequenced with Illumina HiSeq 2000 technology. Read pairs obtained for each strain were filtered and assembled into 44,983 and 44,564 transcripts for Tiso-Wt and Tiso-S2M2, respectively (Table 1). These two datasets were clustered to obtain a final consensus transcriptome of 46,687 transcripts. Of these, 34,374 were unique, representing a total length of 44.4 Mb. At least two isoforms were detected for the remaining (12,313) transcripts. The origin of these non-unique transcripts could be the sequencing of mature and non-mature RNA, alternatively spliced or the sequencing of duplicated genes [27]. The consensus transcriptome was annotated and 14,790 transcripts were associated with at least one Gene Ontology (GO) term. These transcripts with putative function were sorted according to cellular functions (Figure 1). Globally, enzymes of all major metabolic pathways such as nitrogen, photosynthesis or sugar metabolisms were represented and among those, 2,010 transcripts were assigned at least one known universal KEGG pathway (Kyoto Encyclopedia of Genes and Genomes, http://www.genome.jp/kegg/). Specifically, all the major enzymes of lipid metabolism were identified in the Tiso transcriptome and match in the universal KEGG lipid pathway (Figure S1). However, no putative function was identified for 57% of transcripts (19,584).

**Table 1:** Tiso transcriptome characteristics.

Libraries	Read pairs, raw data	Read pairs produced with good quality	Assembly transcripts	Consensus transcripts between the libraries	Read pairs aligned on unique *loci*	Read pairs aligned on multi *loci*	Read pairs not aligned
Wt	177 M	140 M (79.1%)	44,983	46,687 among them	100 M (71.6%)	30 M (21.7%)	9 M (6.7%)
S2M2	189 M	156 (82.4%)	44,564	34,374 unique	115 M (73.7%)	30 (19.3%)	6 M (4.0%)

The table summarizes the different steps in building the Tiso transcriptome using RNAseq data obtained from Illumina HiSeq technology.

### Comparison of Tiso transcriptomes with reference microalgal transcriptomes

**Figure 2:**
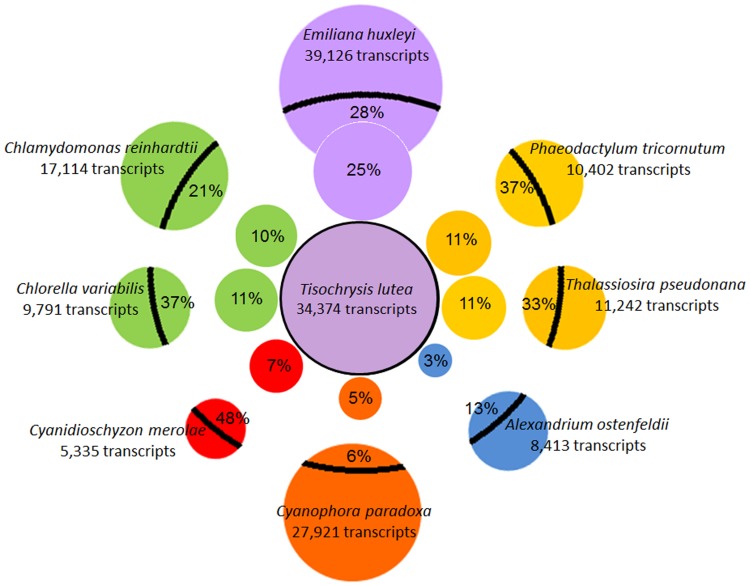
Comparative analysis of consensus Tiso transcriptome with those of reference microalgae. The size of circles represents transcript number. Color coding: green circle for chlorophytes, red for rhodophyte, orange for glaucophyte, blue for dinoflagellate, yellow for diatoms and purple for haptophyte. The number in each peripheral circles show proportion (%) homologous genes with Tiso. The number in each inner circles show proportion (%) homologous genes with reference microalgae.

The consensus Tiso transcriptome was compared with transcriptomes from eight reference microalgal taxa chosen for their diverging phylogenetic position (2 chlorophytes, 2 diatoms, 1 rhodophyte, 1 glaucophyte, 1 dinoflagellate and 1 haptohyte). For each algae transcriptome, we considered only the coding transcripts known to produce a putative protein in order to avoid biases due to the existence of non-coding transcripts. The number of transcripts in each transcriptome and the number of homologous genes between Tiso and these reference microalgae are shown in Figure 2. Tiso has many more putative unique transcripts (more than 30,000) with the most closely related species in the list, *Emiliania huxleyi*, than with the other reference microalgae. Unsurprisingly, *E. huxleyi* possesses the highest proportion (25%) of genes that are homologous with those of Tiso, the proportion being less than 11% with the reference microalgae from other lineages. Finally, only 750 genes that were homologous between all of these algae were identified. The high diversity and very ancient divergence of these reference algae [28] explains this low number of homologous genes. Indeed, 66% (22,664) of Tiso genes have not homologous from these microalgae.

Codon usage bias and transcriptomic G+C content were also compared between the reference algae (Figures S2 and S3). The codon usage bias refers to differences in the frequency of occurrence of synonymous codons. Generally, the codon preference reflects a balance between mutations and natural selection for translational optimization [29]. The G+C content in coding DNA is variable between species and is known to play a role in the codon usage bias [30]. Our results indicate that the codon usage bias of Tiso is similar to that of the other haptophyte *E. huxleyi*, but also to that of the dinoflagellate *Alexandrium ostenfeldii* (Figure S3). Surprisingly, Tiso has a transcriptomic G+C content (60.2%) more similar to that of the dinoflagellate A. *ostenfeldii* (58.3%) than that of *E. huxleyi* (Figure S2).

#### Impact of the selection program on the transcriptome of Tiso-S2M2

The Tiso wild type strain is monospecific, but is presumed not to be a clonal strain but composed of several genotypes characterizing a population. During the selection program [22], diversity of Tiso-Wt was not explored. The identification of transcriptome polymorphisms gives some light concerning the molecular diversity of this strain. Moreover, the selection program applied to Tiso was accomplished in closed conditions [22]. Therefore, the polymorphism observed between Tiso-Wt and Tiso-S2M2 was generated only by mutation and/or within-strain selection effects and not by allele flux from the environment. In these conditions, the impact of the selection program on the genetic diversity of Tiso-S2M2 could be studied.

**Figure 3:**
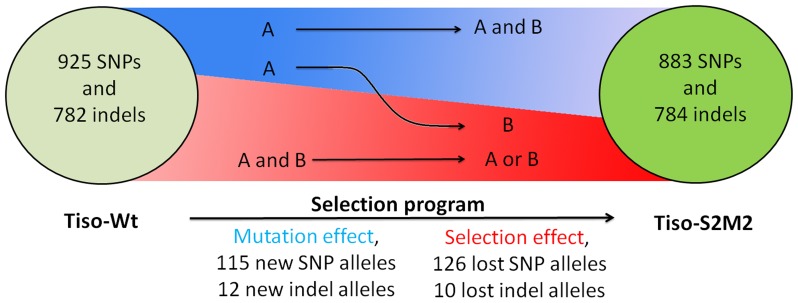
Impact of the selection program on the Tiso transcriptome. This figure is a schematic representation of the selection program. The number in each circle represents genetic diversity for each strain. Between these circles, evolutionary origins of polymorphisms between strains are illustrated: in red, polymorphism generated by loss of alleles (selection events), the narrower of area represent a reduction of diversity; in blue, new alleles (mutation events) the top of the area represents an increase of diversity; the arrow from blue to red, new and selected alleles (mutation and selection events).

**Figure 4:**
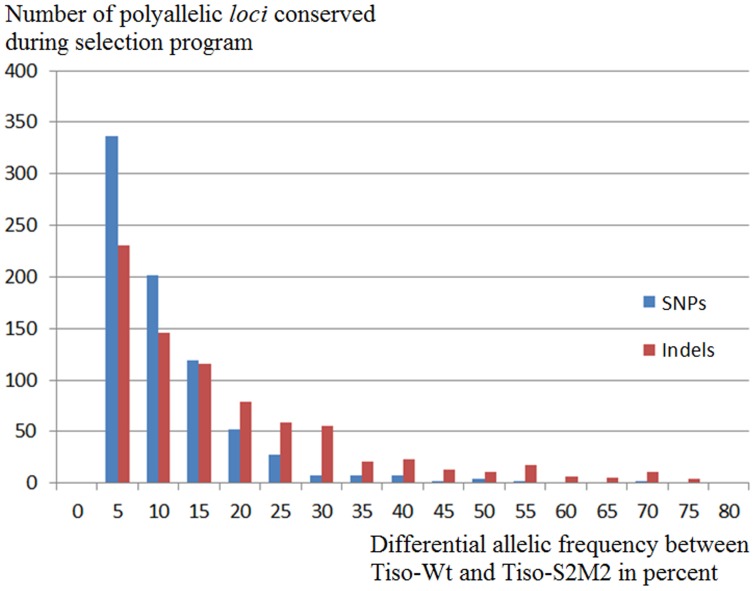
Distribution of differential allelic frequency between Tiso-Wt and Tiso-S2M2. This histogram represents the number of polymorph loci (SNP and indel) following their conservation rate through the selection program. Conservation rate defined as percent represents the differential allelic frequency between Tiso-Wt and Tiso-S2M2.

We first investigated the molecular diversity in each strain at the transcriptome level and its evolution during the selection program. Polyallelic *loci* were identified and analyzed in the transcriptome of each strain (Figure 3) to estimate the molecular diversity. For SNPs, 925 and 883 biallelic *loci* were detected for Tiso-Wt and Tiso-S2M2, respectively. As for the indels, 782 and 784 biallelic *loci* were detected, respectively. These results confirm that both strains are composed of several genotypes. Allelic frequency of polymorph *loci* was compared between both strains to look for theevolution of the genetic diversity during selection program,. Only transcripts without differentially expressional level were considered, in each population. A Jost's D genetic index [31] was used and a score of 0.161 was estimated between both strains showing a high conservation of genetic diversity between the populations. Indeed, the majority of polyallelic *loci* detected in Tiso-Wt, i.e. 798 (86%) SNPs and 762 (97%) indels, were conserved in Tiso-S2M2. For each conserved polyallelic *locus*, the differential allelic frequency between the two strains was measured (Figure 4). On average, the differential allelic frequency per *locus* was 15.6% for SNPs and 8.5% for indels confirm a global conservation of genetic diversity at the end of the selection program. 92 SNPs and 20 indels were *loci* with a differential allele frequency greater than 35% (Figure 4), reflecting selection events during the selection program. Five transcripts containing more than five polyallelic *loci* with a differential allele frequency are of particular interest, these being considered as transcripts with a clear selection signature.

Thereafter, we focused on the positional polymorphisms and differentially expression levels generated during the selection program. For positional polymorphisms, 241 SNP and 22 indel polymorphs were detected between the strains. SNP and indel polymorphisms were distributed among 146 and 19 transcripts, respectively (Figure S4). The large majority of these transcripts (124/165) contained only one polymorphism. However, seven hot spots of polymorphism with more than five polymorphisms in a specific region of one transcript were identified (frequency superior to 1 polymorphism per 100 bases). Among these seven hot spots, four were localized in UTR regions and three in coding regions.

Concerning differential gene expression levels, 84 transcripts were over-expressed and 207 under-expressed in Tiso-S2M2 compared to Tiso-Wt. Of these, 32 had a high expression difference (>100 fold) and 18 transcripts showed a specific expression in only one strain (Table S1). More precisely, eleven transcripts were specifically expressed in Tiso-S2M2 and seven transcripts were specific to Tiso-Wt. These results show that expression of some genes was clearly affected as a result of the selection program.

#### Origin of polymorphisms

The origin of polymorphisms (mutation or selection events) was identified to evaluate the evolution strength producing from the selection program. The selection program was conducted in closed conditions and consequently the 241 SNP and 22 indel polymorphisms between the strains could be sorted into different classes (Table 2). Class 1 comprises the polymorphisms for which there was one allele in Tiso-Wt and two alleles in Tiso-S2M2 (Figure 3). This class refers to polymorphisms generated by mutation events occurring during the selection program (85 SNPs and 12 indels; Table 2). Class 2 comprises polymorphisms for which there were two alleles in Tiso-Wt, only one of which was conserved in Tiso-S2M2 (Figure 3). This implies a loss of one allele by selection pressure (126 SNPs and 10 indels; Table 2). Finally, class 3 comprises polymorphisms for which there was one allele in Tiso-Wt and a different allele in Tiso-S2M2 (Figure 3). This refers to polymorphisms appearing as a result of mutation followed by selection (30 SNPs and 0 indels; Table 2).

**Table 2:** Genotype class of polymorphic *loci* between wild type Tiso-Wt and selected Tiso-S2M2.

Class	Tiso-Wt		Tiso-S2M2	Polymorphism number
1	*Locus* A	= >	*Locus* A/B	85 SNPs
2	*Locus* A/B	= >	*Locus* A or B	126 SNPs
3	*Locus* A	= >	*Locus* B	30 SNPs

Class 1 shows polymorphisms generated by a mutation event. Wild allele (A) and mutated allele (B) were observed in Tiso-S2M2. Class 2 shows polymorphisms generated by selection events only. Only one allele, (A) or (B) was observed in Tiso-S2M2 strain, whereas the two alleles were found in Tiso-Wt strain. Class 3 shows polymorphisms generated by both mutation and selection events. Only the allele (B) was detected in Tiso-S2M2.

#### Annotation and selection of candidate genes for lipid over-accumulation

**Figure 5:**
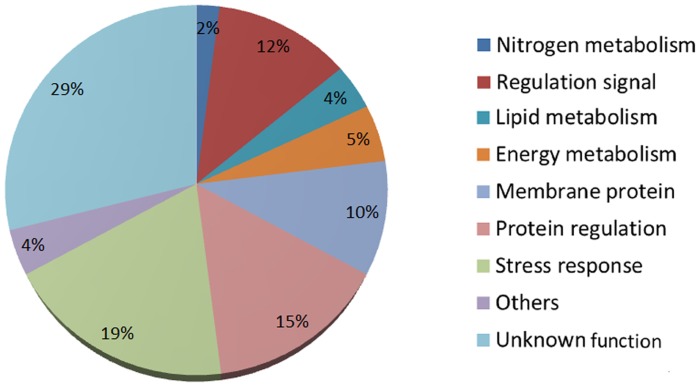
Expert annotations of transcripts containing polymorphisms or differential gene expression between Tiso-Wt and Tiso-S2M2. Transcripts were sorted according to major cell function.

Transcripts with position polymorphism or differentially expressed between Tiso-Wt and Tiso-S2M2 were manually annotated and sorted according to metabolic pathways (Figure 5). A large number of differentially expressed transcripts involved in stress responses, protein signal regulation and membrane proteins (transporters and structural proteins) were identified. Few genes were classed as being involved in energy, lipid or nitrogen metabolism.

The selected strain Tiso-S2M2 is characterized by an over-accumulation of lipids in nitrogen-limited culture conditions and we focused on this aspect. Candidate genes were identified by a combined analysis of polymorphism between the strains. Firstly, transcripts with a selection signature generated by the impact of the selection program could be interesting candidates. Among positional polymorphisms, three hot spots located in coding regions of *TisoTranscripts-291*, *TisoTranscripts-227* and *TisoTranscripts-46* were detected (21, 7 and 6 SNPs were identified for each transcripts respectively). These hot spots contain polymorphisms only produced by selection effects and consequently are considered as transcripts with a selection signature. Moreover, some polymorphisms (12, 3 and 1 for *TisoTranscripts-291*, *TisoTranscripts-227* and *TisoTranscripts-46* respectively) were non synonymous and could induce a change of protein activities. Annotation of these candidate genes revealed a putative Na^+^ solute transporter (*TisoTranscripts-291*) and two proteins with zinc finger domains (*TisoTranscripts-227* and *TisoTranscripts-46*). One homologous gene of *TisoTranscripts-291* was exclusively identified in *E. huxleyi* (*Emihu-233786*) suggesting a haptophyte specific gene. Five transcripts with hot spots of *loci* with differential allelic frequency were also detected (*TisoTranscripts-440*, *TisoTranscripts-441*, *TisoTranscripts-442*, *TisoTranscripts-443* and *TisoTranscripts-444*), but no function was found for these candidate genes bearing a selection signature.

In the same ecophysiological conditions, a total of 298 transcripts were differentially expressed between the two strains. Gene expression was therefore clearly affected by the selection program and this could explain lipid over-accumulation in Tiso-S2M2. A total of 18 transcripts were expressed specifically in only one strain (Table S1) and these were considered as promising candidate genes for further studies. The putative annotation of only three of these genes was possible. *TisoTranscripts-9* was positively assigned as a putative transcription factor, *TisoTranscripts-57* as an ankyrin protein-like and *TisoTranscripts-59* a Glycosyl-PhosphatIdylinositol protein-like (GPI protein). No homologous genes of these candidates were identified in reference microalgae suggesting that these candidates are specific to Tiso.

Because the selected Tiso-S2M2 strain is characterized by lipid over-accumulation, the transcripts with a putative function linked to lipid metabolism and with positional polymorphism or differential gene expression were considered as good candidates. *TisoTranscripts-288* and *TisoTranscripts-160*, annotated as a long chain fatty acid ligase and a GDSL lipase respectively, showed differential expression (under expressed 53- and 24-fold in TisoS2M2, respectively). Their respective expression was confirmed by a RT-qPCR experiment (Data S1) and we consider them as excellent candidates for future studies. Moreover, homologous gene of *TisoTranscripts-288* was identified in *E. huxleyi* (*Emihu-456684*), *P. tricornutum* (*Phatr-45510*) and *C. merolae* (*CMG-147C*). However, *TisoTranscripts-160* was only one homologous identified in *E. huxleyi* (*Emihu-213608*).

## Discussion

The haptophyte *Isochrysis* affinis *galbana* is a species of major economic importance in aquaculture with good potential for biofuel production [12]. A laboratory selection program was developed and a strain obtained (Tiso-S2M2) that accumulates more lipid in nitrogen-limiting conditions than the wild type [22]. Molecular evolution of Tiso-Wt resulting from the selection program was studied and this allowed us to establish a list of candidate genes that could play a role in lipid over-accumulation. Moreover, fine-scale analysis of the transcriptomes of the wild type and selected strains provides new insights concerning the life cycle of Tiso.

### First molecular knowledge of Tiso

In this study, transcriptomic data was produced for Tiso for the first time using high-throughput sequencing. The transcriptomes were annotated and all major metabolic pathways were identified. Annotation of these major pathways is usually relatively easy because they contain highly conserved protein domains [32]. However, no putative function was found for 57% of transcripts. To date, the number of genes with known function is very low for microalgae and the high diversity of microalgae makes annotation difficult [14].

The consensus transcriptome of Tiso was compared with eight other transcriptomes from reference microalgae. These microalgae were chosen because of their phylogenetic position and the availability of transcriptomic data. We compared the total number of transcripts and homologous genes, the transcriptomic G+C content and the codon usage bias. In general and as expected, these results confirmed that Tiso is genetically very closely related to *Emiliania huxleyi*, which is a member of the same haptophyte order, the Isochrysidales. Surprisingly, the codon usage bias and transcriptomic G+C content of Tiso were more similar to those of the dinoflagellate *Alexandrium ostenfeldii* than to those of *E. huxleyi*. Although these characteristics are not significant in an evolutionary sense, they reflect a high level of genomic speciation since the divergence between Tiso and *E. huxleyi*, estimated around 120 Mya [33], [34].

### Transcriptome evolution as a result of the selection program

The lipid over-accumulating selected strain Tiso-S2M2 was obtained from the wild type strain by a selection program [22]. This selection program consisted of increasing the genetic diversity by UVc exposition followed by selection of lipid-rich cells by flow cytometry.

In this study, we measured and analyzed the molecular modifications produced by the selection program on the population of Tiso-Wt at transcriptome level. These results could help to refine future selection programs. To measure the impact of this selection program, the genetic diversity of Tiso-Wt and Tiso-S2M2 was evaluated and compared at the transcriptome level. Polyallelic *loci* identified for both strains confirm the non-clonal character of Tiso-Wt and Tiso-S2M2. Interestingly, polyallelic *loci* detected in Tiso-Wt were mostly conserved in Tiso-S2M2 and their allele frequency did not change significantly after the selection program.

RNAseq strategy allowed us to analyze the gene expression and the positional polymorphism. Differential gene expression level observed for almost any cell functions reveals important modifications in all metabolism pathways. Studies of the inter-genic regions and the large polymorph insertions (requiring genome sequencing approaches) are underway. In turn, positional polymorphisms on coding region were observed between both strains. Among these polymorphisms, SNP mutations were more frequently detected than indels (i.e: 242 SNPs and 22 indels). This could be explained by a stronger selection pressure on indels than SNPs. Indeed, it is known that indel polymorphism has a stronger impact on phenotype because indel mutations are more harmful [35].

The selection program was undertaken in non-clonal strain in closed conditions and consequently the polymorphism observed between the strains was generated only by mutation or selection [22]. We sorted polymorphisms (Figure 3) according to whether they were: (i) new alleles generated by mutation events (class 1 and class 3, Table 2); (ii) loss of alleles in Tiso-S2M2 by a selection effect (class 2, Table 2). It is noteworthy that the numbers of new and lost alleles were approximately equal. This suggests that mutation and selection strengths were balanced [36], [37]. The selection program was thus characterized by maintenance of genetic diversity [38], which has the theoretical advantage of producing a strain with higher capacity to adapt to environmental variability compared to a clonal strain [39]. The lipid over-accumulating phenotype in Tiso-S2M2 has been conserved in the absence of selective pressure about lipid contents for more than two years [22]. This could be explained by the selected trait being more easily maintained in a population with high genetic diversity [40], [41].

The clonal diversity of Tiso-S2M2 will be used in a next program to further select lipid accumulating Tiso strains. Two selection strategies will be investigated: One will be to select the best clone for lipid content from the Tiso-S2M2 population. However, conservation of clonal strains is usually risky on a long term basis and cryopreservation could be the solution [42]. Another selection strategy could call for a different mutagenesis methods and higher selection pressure.

### List of candidate genes for lipid over-accumulation in the selected Tiso strain (Tiso-S2M2)

The selection program modified globally all cell functions (Figure 5). The selection of lipid trait generated large modifications in cells. These large modifications could be explained by selection program not affecting only lipid trait but, for example, selection of cells with UVc tolerance. Furthermore, the lipid metabolism is in relationship with all other metabolisms and a weak modification can affect entire cells. We focused on the lipid metabolism as biofuel production by microalgae is considered as one of the most promising sources for future energy production [9]. Identification of genes or alleles that play roles in lipid over-accumulation could be of great interest for future selection and metabolic engineering programs. A combined approach was used to produce a list of candidate genes that included: (i) transcripts with a selection signature; (ii) transcripts with specific expression in only one of the strains; (iii) transcripts with a putative function in lipid metabolism and with a positional polymorphism or differential gene expression.

In this study, eight transcripts with a clear selection signature were detected. Of these, only three could be annotated. Two of these genes (*TisoTranscripts-227* and *TisoTranscripts-46*) encode proteins with zinc finger domains which are known to play an important role in the regulation of gene expression [43]. The target genes of these two candidates are not currently known, but their selection signatures imply a role in increasing the fitness of Tiso-S2M2 in terms of lipid accumulation or UVc tolerance. No homologous genes were identified in reference microalgae suggesting a specific regulation of Tiso. According to the annotation, the third transcript (*TisoTranscripts-291*) is a Na^+^ solute transporter-like. The most closely related protein domain that we could identify is the SLC5 family [44]. This family of co-transporters is known to exchange Na^+^ solutes with different substrates such as glucose, urea or amino acids [45]. Allele selection of this gene could allow Tiso-S2M2 to optimize the transport of a molecule involved in the resistance to the selection program. A functional study of these genes would be of great interest to understand their role in the Tiso-S2M2 phenotype and specifically in lipid metabolism.

Gene expression was affected by the selection program. Of the 34,374 transcripts of Tiso, 291 exhibited differential expression between the strains. This differential gene expression could have several origins, such as mutations in regulation regions or epigenetic variations. Of these transcripts, 18 were specifically expressed in Tiso-Wt or Tiso-S2M2. With the highest differential expression, these are considered as serious candidates to explain the selection result. Other genes with differential expression could also be good candidates, but their differential expression need to be confirmed by a qRT-PCR approach [46]. Among the 18 main differentially expressed candidates, a putative function was found for only three genes. These candidates as well have no homologous genes in reference microalgae. *TisoTranscripts-9* encodes a putative transcription factor. Differential expression of this type of gene is known to modify expression of target genes and thus metabolic pathways. *TisoTranscripts-57* codes for an ankyrin protein-like. The ankyrin domain is considered to be the most common location for protein–protein interactions [47] and can play a role in several cellular functions. *TisoTranscripts-59* was identified as a GPI protein-like, a category of proteins known for their roles in communication between cells [48]. Given their putative functions, a potential implication of these latter two genes in the lipid over-accumulation observed in Tiso-S2M2 is not obvious, but they should be kept in mind for further studies.

The targeted phenotypic character of the selected strain is lipid over-accumulation and we therefore focused on the annotated genes of lipid pathways. Transcripts with putative annotation linked to lipid metabolism and containing a positional polymorphism or differential expression level were selected. Two candidate genes were identified (*TisoTranscripts-288* and *TisoTranscripts-160*) with differential expression between the strains. These published results rely on RNAseq dataset and because the implementation of this technique is however rather new, we confirmed the results using the well-established RT-qPCR technique (data S1) in a biological replicates. *TisoTranscripts-288* was identified as encoding a putative long chain fatty acid ligase (ACLS, EC: 6.2.1.3). This enzyme family is known to esterify free fatty acids containing C14/C20 carbon chains into fatty acyl-coenzyme A (acyl-CoA) [49], [50]. Esterification into fatty acyl-CoA is a key step in numerous lipid metabolism pathways, in particular those involved in lipid catabolism. Analysis of the Tiso transcriptome revealed that *TisoTranscripts-288* was the only transcript annotated as coding for ACLS proteins. Consequently, the under-expression of this enzyme in Tiso-S2M2 may suggest a defect in lipid catabolism causing the lipid over-accumulation phenotype. Functional study of this enzyme is under way and will allow testing this hypothesis. The other candidate gene (*TisoTranscripts-160*) is assigned as a putative GDLS lipase. This family is composed of hydrolytic enzymes with multifunctional properties such as broad substrate and region specificities [51]. Two other GDSL lipase transcripts were identified in the Tiso transcriptome, but these were not differentially expressed. Functional study of the under-expressed GDLS lipase is also required to determine whether it is implicated in lipid catabolism. Surprisingly, the expression of known and identified genes involved in either fatty acid or triacyglycerol biosynthesis was similar between the two strains. In contrast, these candidate genes (*TisoTranscripts-288* and *TisoTranscripts-160*) showed the catabolic pathway related to fatty acid oxidation and hydrolytic lipase activity appears to have been affected.

### Insights into the life cycle of Tiso

Many haptophytes, including *Emiliania huxleyi*, are known to undergo dimorphic haplo-diplontic life cycles, in which both haploid and diploid phases are capable of independent asexual division [15]. Ploidy level and sexual stages have never been reported for Tiso, or for any other member of the Isochrysidaceae. In culture, isochrysidaceaens have a single non-calcifying morphotype that resembles the haploid phase of noelhaerhabdaceaens (i.e. non-calcifying, usually flagellate). Diploid phase calcification is thought to have evolved only once at the origin of coccolithophores, and was thus apparently lost early in the evolutionary history of the Isochrysidaceae [52]. It is not clear whether this reflects a complete loss, or reduction, of the diploid phase in these species (i.e. clonal or haplontic life cycle, respectively), or whether they undergo a haplo-diplontic life cycle like other haptophytes, but with isomorphic non-calcifying haploid and diploid stages.

Knowledge of ploidy level is the basis for the understanding of genetics of species [53] and knowledge of the life cycle of this microalga could allow addition of breeding steps during the selection program [18]. This type of information would enable, for example, the study of heritability, gene functions or regulation, and allele interactions. Study of the evolution of genetic diversity resulting from the selection program and observation of evolutionary origin for positional polymorphisms provides insights into the life cycle of Tiso in laboratory conditions.

Selection events typically generate a loss of genetic diversity in populations [54]. Numerous (136) alleles present in Tiso-Wt were selected in Tiso-S2M2 (class 2, Table 2) and several (30) new alleles that appeared as a result of mutation were also selected in Tiso-S2M2 (class 3, Table 2). The majority of genetic diversity observed in Tiso-Wt was conserved in Tiso-S2M2. The conservation of the observed diversity despite the selection strength necessarily suggests allelic recombination during the selection program. We suggest that this allelic recombination implies a sexual step during our selection program. In other microalgae, such as diatoms [55], sexual reproduction has also been observed in response to particular environmental conditions [56], [57]. Observation and physiological conditions of the fertilization step in Tiso will be confirmed in future studies.

Evolutionary origins of positional polymorphisms were analyzed to determine the ploidy of Tiso. Polymorphisms were sorted into three classes (Table 2). Because we observed multiallelic *loci* in both strains, the first proposed hypothesis is that Tiso is diploid. In this case, class 1 and class 2 polymorphisms (Table 2) could be explained by a mutation event on one allele (class 1) or selection events which had selected one of the two alleles in Tiso-Wt (class 2). However, for the third class, the origin of polymorphisms is, in this case, difficult to explain. The first possibility is a mutation event on each allele at the same *locus* and selection of both mutations in TisoS2M2. The probability of a mutation impacting the two alleles at the same *locus* is near null (P = 1/(number of nucleotides in genome)^2^). Consequently, this hypothesis is not conceivable. A second possibility is that a mutation appears on one allele and this individual undergoes self-fertilization. A part of this progeny is homozygote for the new allele and can be selected. However, in this case a high loss of genetic diversity in Tiso-S2M2 would be expected, which was not the case. These considerations lead us to suggest that Tiso is not diploid during the major stage of its life cycle, but rather that the Tiso-Wt and Tiso-S2M2 strains are composed of haploid cells. This latter hypothesis is attractive because it can explain the presence of polymorphisms in the third class by one mutation and one selection event.

The Tiso strains appear thus to be haploid in our culture conditions and capable of sexual reproduction, which means they either have a haplontic or a haplo-diplontic life cycle. Isolation and crossing of clonal haploid cultures could therefore be feasible in the context of development of a breeding program.

## Conclusion

This study brings molecular basis of *Tisochrysis lutea* in a perspective of intensive selection program but complementary studies such as genome sequencing project or large study of genetic diversity will be conducting. Here, we added value to a previous program by analyzing at the genetic level the events provoked by the mutation/selection procedure. This analysis shows that balanced selection led to production of a strain that over-accumulates lipids. A comparative analysis of polymorphisms in the strains allowed identification of 8 genes that are candidates for involvement in provoking this phenotypic difference. These are promising targets for functional studies in the perspective of developing marker-assisted selection and genetic engineering protocols.

## Materials and Methods

### Microalgae strains and culture conditions

*Isochrysis* affinis *galbana* recently renamed *Tisochrysis lutea* clone Tahiti (Tiso) [11] was provided by the Culture Centre of Algae and Protozoa (CCAP 926/14). This Tiso wild type strain is monospecific and was isolated by Haines in the late 70s and kept in algae bank until today. Tiso wild type strain has been used in the selection program previously realized in our laboratory [22] and is considered as the reference strain in this study (Tiso-Wt). The selection program allowed to acquire a new strain (Tiso-S2M2) with higher lipid content than Tiso-Wt in nitrogen starved conditions [22]. This selected strain was certificate (IFR 32B85, [26]). Strains were grown in 2 L flasks containing modified Conway medium [57] with a modified nitrate concentration of 0.12 mM and bubbled with 0.22 µm filtered-air. Cultures were maintained at a constant temperature of 21°C and under constant irradiance of 100 µmol m^−2^ s^−1^. The harvesting of microalgae was undertaken at the same time of day for both strains at the onset of nitrogen starvation when Tiso-S2M2 over-accumulated more lipids than Tiso-Wt (Figure S5).

### RNA extraction, cDNA library construction and sequencing

Total RNA was extracted from each strain (Tiso-Wt and Tiso-S2M2) using the TRIZOL reagent (Invitrogen, USA) according to the manufacturer's instructions. DNase treatment (DNase RQ1, Promega) was used to remove residual genomic DNA. The quality and quantity of purified total RNA were determined by measurement of absorbance (260 nm/280 nm) using a Nanodrop ND-1000 spectrophotometer (LabTech, USA). Poly(A) mRNA was isolated from total RNA using oligo(dT) magnetic beads (MicroPoly(A)PuristTM Kit, Ambion) according to the manufacturer's instructions. The first and second-strand cDNA synthesis was performed on purified mRNA using the SuperScript Double-Stranded cDNA Synthesis Kit (Invitrogen, USA) according to the manufacturer's protocol. The two cDNA libraries were constructed and sequenced with an Illumina HiSeq 2000 sequencer (Illumina Corporation Inc.). Approximately 4–5 µg of cDNA were used for library construction, undertaken by the Genoscope platform (http://www.genoscope.cns.fr). Sequencing was performed using the paired-ends method with a read length of 100 bases, producing an average of 183 Million of read pairs per transcriptome. Read pairs obtained were analyzed with FastQC software developed by S. Andrews in the Babraham Institute (www.bioinformatics.bbsrc.ac.uk) in order to validate run qualities (read number, quality score of nucleotide sequenced in Q-phred scale [58], composition of reads). We sequenced 35 and 37 Gb for Tiso-Wt and Tiso-S2M2 cDNA libraries respectively (Table 1) with a mean sequencing quality score per read at Q35. Sequence data for this article have been deposited in the National Center for Biotechnology Information and are accessible in: SRR823264 for Tiso-S2M2 and SRR824147 for Tiso-Wt.

### Construction of the reference transcriptome for Tiso

Read pairs obtained for each sample were filtered to select only read pairs with the correct quality to be assembled. First, reads containing Illumina sequencing adapter and Illumina control sequences were eliminated with CutAdapt software [59]. In a second step, reads were filtered on a quality score of the last nucleotides for each read because quality of sequencing decreases proportionally with read length [60], [61]. The last nucleotides were eliminated until detection of a nucleotide with a quality score of Q25. In a third step, reads were filtered on their length. Reads inferior to 75 bp were eliminated. In a final step, reads were filtered on a mean quality score of all nucleotides. Reads with a quality score inferior to Q25 were eliminated. After these different screens, 79.1% and 82.4% total read pairs for Tiso-Wt and Tiso-S2M2 respectively were conserved (Table 1).

Read pairs of each library were assembled with the Trinity pipeline [62] with the parameters advised by Zhao Q-Y *et al.*, [63]. The putative transcripts assembled for each library were clustered with CD-hit-EST software with 90% identity [64] to obtain the consensus transcripts considered as the reference transcriptome of Tiso (Table 1). Transcriptome datasets of each strain (Tiso-Wt and Tiso-S2M2) were aligned using the reference transcriptome with MosaikAssembler software (Wan-Ping Lee and Michael Strömberg, Marth lab). On average, 72.7% (σ = 1.4) read pairs were aligned on unique *locus*, 20.5% (σ = 1.6) aligned on multiple *loci* and 5.4% (σ = 1.9) were not aligned (Table 1). Transcripts composed at 90% of reads aligned in multiple *loci* were considered as genes with 2 or more isoforms.

### Research of homologous genes from several reference microalgae and comparison of codon bias

Transcriptome data from Tiso were compared with data from 8 other reference microalgae. Data for 5 of these (the haptohyte *Emiliana huxleyi* [65], the diatoms *Phaeodactylum tricormutum* [66] and *Thalassiosira pseudonana* [67], the chlorophytes *Chlorella variabilis* [68] and *Chlamydomonas reinhardii* [69]) were produced by the US Department of Energy Joint Genome Institute (JGI http://www.jgi.doe.gov/) and are publically available in the web site. The other reference microalga used were the dinoflagellate *Alexandrium ostenfeldii* [70], the glaucophyte *Cyanophora paradoxa* [71] and the rhodophyte *Cyanidioschyzon merolae* [72]. Homologous transcripts were detected by BLAST analysis [73] between the transcriptome data of reference microalgae and Tiso (tblastx with alignment length greater than 100 amino acids and identity score greater than 30%). Codon usage bias was calculated with the Sequence Manipulation Suite software [74] on coding regions of each reference taxon. For Tiso, the putative coding regions of each transcript were identified with ORF-predictor [75]. A similarity tree of codon bias was built from correlation scores obtained between microalgae and drawn with the Darwin software (http://darwin.cirad.fr/) using the hierarchical clustering method WPGMA [76].

### Annotation of Tiso trancriptome

Research of putative function was undertaken by BLAST analysis [73] on the NCBI database (nr bank; blastx with alignment length greater than 100 amino acids and identity score greater than 30%). Transcripts were sorted automatically for major cell functions. For the transcripts showing a polymorphism between Tiso-Wt and Tiso-S2M2, expert annotation was done. A second BLAST on SwissProt database (v. May2012) and functional domain was performed with InterProScan [77]. Consensus of annotation results was manually attributed for putative function for each transcript.

### Differential expression analysis

After read alignment for each strain on the reference transcriptome, to search differential expression we normalized the libraries themselves as a function of mean depth per putative transcript (324× for Tiso-Wt and 389× for Tiso-S2M2). The number of read pairs aligned per transcript was compared between strains. Differential expression was considered if the difference between the 2 strains was up to 20-fold (log2) and if for both the depth was superior to 50 reads. Differential expressions were also confirmed with the recent GFOLD algorithm [78] with the p-value fixed at 0.001.

### Positional polymorphism identification and estimation of genetic diversity

Positional polymorphisms, i.e. Single Nucleotide Polymorphism (SNP) and short insertion/deletions (indel), were identified between transcripts of Tiso-Wt and Tiso-S2M2. We used Freebayes software (Erik Garrison, Marth lab) and validated polymorphisms if depth was superior to 50× for each individual and if a minority of alleles had a depth superior to 10×.

Genetic diversity was measured for each strain (Tiso-Wt and Tiso-S2M2). Polyallelic *loci* were detected in transcriptomes of each strain. A *locus* was considered polyallelic if the depth of reads was greater than 50×. For each polyallelic *locus*, frequency of each allele was measured (number of alleles divided by sum of total alleles). Comparison of global genetic diversity between the both strains was estimate with D of Jost [31] using SPADE program [79].

Sequence data for this article have been deposited in the National Center for Biotechnology Information and are accessible in: SRR823264 for Tiso-S2M2 and SRR824147 for Tiso-Wt.

## Supporting Information

Data S1 **Methods and results of RT-qPCR approach for the candidates** ***TisoTranscripts-288*** **and** ***TisoTranscripts-160*** **.**
(DOC)

Figure S1 **Enzymes of lipid pathways identified in universal KEGG.** Each enzyme with a colour corresponds to a transcript annotated in KEEG. Figure A corresponds at lipid synthesis and Figure B catabolism of lipid.
(TIF)

Figure S2 **Comparison of G+C content in transcriptomes of Tiso and reference microalgae.** Colour code: green for chlorophytes, red for rhodophyte, orange for glaucophyte, blue for dinoflagellate, yellow for diatoms and purple for haptophytes.
(TIF)

Figure S3 **Codon bias analysis.** Figure 3A shows codon bias of Tiso and 3B shows comparison of codon bias between reference microalgae. The tree was built using a correlation score matrix of codon bias between microalgae with the hierarchical clustering method WPGMA. Color code: green for chlorophytes, red for rhodophyte, orange for glaucophyte, blue for dinoflagellate, yellow for diatoms and purple for haptophytes.
(TIF)

Figure S4 **Distribution of positional polymorphisms (SNPs and indels) between Tiso-Wt and Tiso-S2M2 per transcripts.**
(TIF)

Figure S5 **Growth of Tiso strains and lipid accumulation.** Cells were counted with a Malassez counting cell and image analysis (SAMBA software). Lipid accumulation was estimated by measurement of Nile Red fluorescence by spectrofluorimetry as described by Bougaran *et al.*, [22]. Red color for Tiso-Wt and black color for Tiso-S2M2. The yellow color shows sampling events.
(TIF)

Table S1 **List of transcripts contained polymorphism identified between Tiso-Wt and Tiso-S2M2.**
(XLSX)
