# Supplementary material for: Comparative Transcriptome of Wild Type and Selected Strains of the Microalgae Tisochrysis lutea Provides Insights into the Genetic Basis, Lipid Metabolism and the Life Cycle
Source: PLoS One. 2014 Jan 29;9(1):e86889. doi: 10.1371/journal.pone.0086889 (PMC3906074; doi:10.1371/journal.pone.0086889)
Supplement: Data S1 — Methods and results of RT-qPCR approach for the candidates TisoTranscripts-288 and TisoTranscripts-160 . (DOC) [file pone.0086889.s001.doc]

**Validation of selected candidate genes by qRT-PCR**

Among selected candidate genes, two genes involved in lipid pathway have an expression differential between Tiso-Wt and Tiso-S2M2. Their differential expression have been validated as well as the accuracy of RNAseq in experiment by RT-qPCR comparisons

Total RNA was extracted from cells using the TRIzol method and then treated with RQ1 DNase to avoid DNA contamination and finally purified using the RNeasy mini kit. cDNA templates for PCR amplification were synthesized from 1 µg of total RNA using the High Capacity cDNA reverse transcription kit (Invitrogen). Quantitative PCR was performed using Power SYBR® Green I PCR master mix in a final volume reaction of 25 μl. All of the reactions were performed following the instructions of the manufacturer with 5 μl of diluted cDNA (1/10) and 0.1 μm of specific primers. Quantitative measurements were performed in duplicate with a Stratagene Mx3000P^TM^ Q-PCR system. The cycling parameters were one cycle of 10 min at 95 °C, followed by 40 cycles of 30 s at 95 °C and 60 s at 60 °C. The results were represented as the relative gene expression normalized to reference genes encoding the elongation factor (Ef1α). Specific primers of TisoTranscript-288 and TisoTranscript-160 transcripts were designed with Primer3Plus program (<http://primer3.sourceforge.net/> ; Table 1). The relative gene expression analysis of candidate genes was performed using the comparative C_T_ method [1].

**Results**

We confirm differential between Tiso-Wt and Tiso-S2M2 in nitrate stress condition for Tisotranscripts-288 and Tisotranscripts-160. Tisotranscripts-288 and Tisotranscripts-160 is under-express (log3 and log7 fold) in Tiso-S2M2 than Tiso-Wt respectively.

| **Transcripts** | **Primer sequence** | **Tm in °C** |
| --- | --- | --- |
| *Tisotranscripts-288* |  |  |
| Left primer  Right primer  Product size: 265 | CAAGGAGGCTATGCAAGAGG  GATGTCCAACGAGGCAAAGT | 59.97  60.12 |
| *Tisotranscripts-160* |  |  |
| Left primer  Tight primer  Product size: 183 | GCTCGCTTGGTAAAATGCTC  CATTATGTCGGTGGTCGATG | 59.99  59.80 |

Table 1. Primers for qRT-PCR

1. Livak KJ, Schmittgen TD: Analysis of relative gene expression data using real-time quantitative PCR and the 2(-Delta Delta C(T)) Method*. Metho*ds 2001, 25:402–8.
