# Supplementary figures and images for: Comparative Transcriptome of Wild Type and Selected Strains of the Microalgae Tisochrysis lutea Provides Insights into the Genetic Basis, Lipid Metabolism and the Life Cycle
Source: PLoS One. 2014 Jan 29;9(1):e86889. doi: 10.1371/journal.pone.0086889 (PMC3906074; doi:10.1371/journal.pone.0086889)

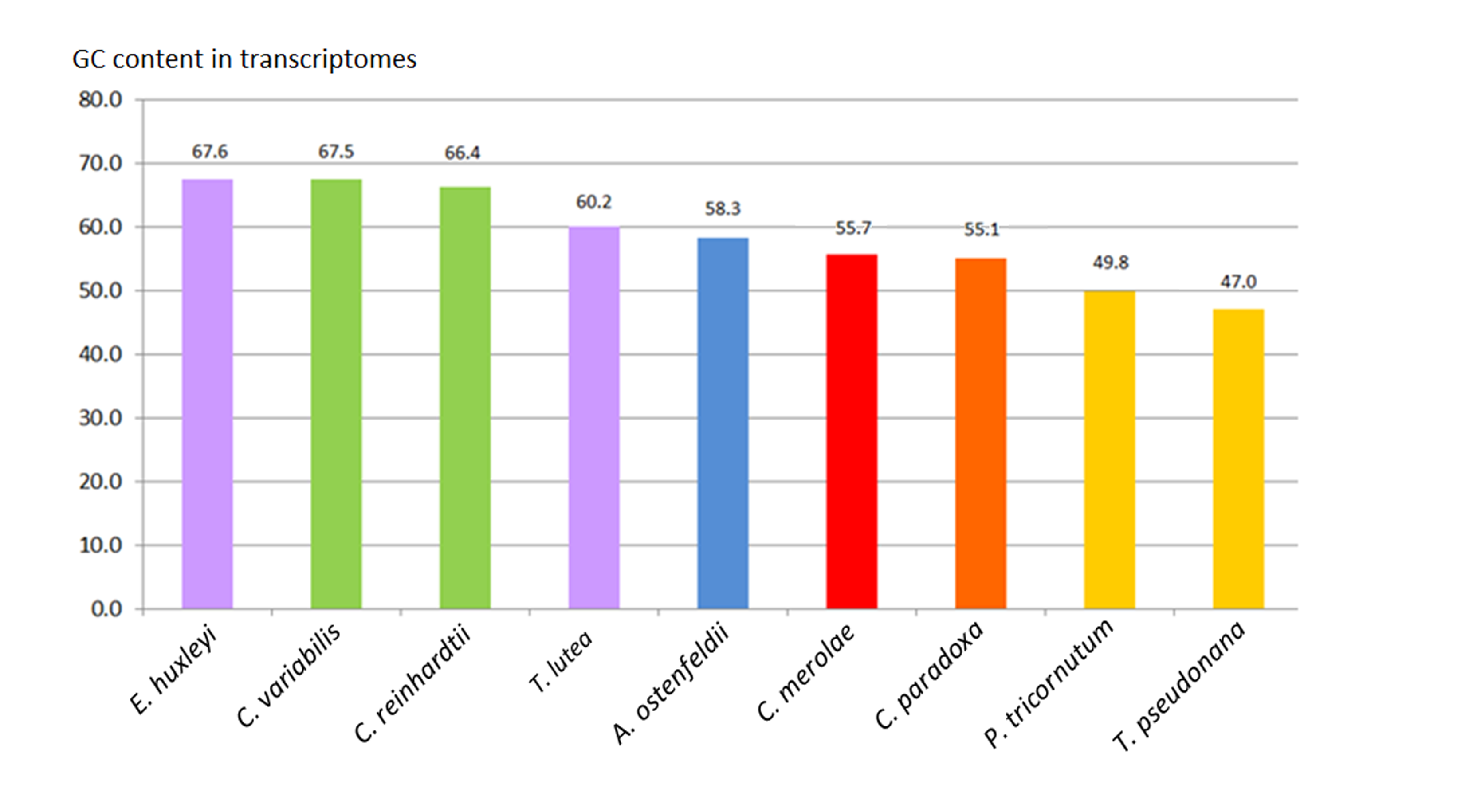

Supplement: Figure S2 — Comparison of G+C content in transcriptomes of Tiso and reference microalgae. Colour code: green for chlorophytes, red for rhodophyte, orange for glaucophyte, blue for dinoflagellate, yellow for diatoms and purple for haptophytes. (TIF) [file pone.0086889.s003.tif]

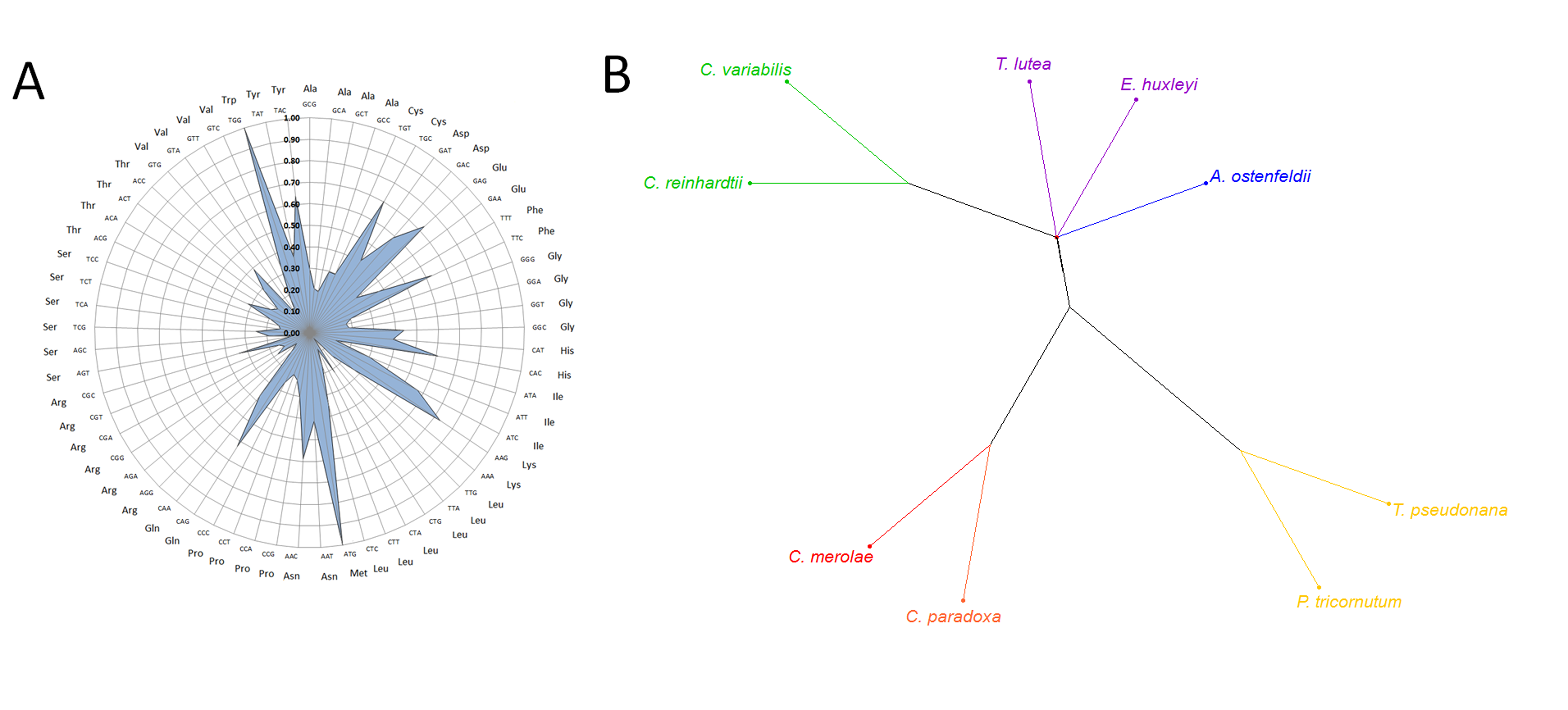

Supplement: Figure S3 — Codon bias analysis. Figure 3A shows codon bias of Tiso and 3B shows comparison of codon bias between reference microalgae. The tree was built using a correlation score matrix of codon bias between microalgae with the hierarchical clustering method WPGMA. Color code: green for chlorophytes, red for rhodophyte, orange for glaucophyte, blue for dinoflagellate, yellow for diatoms and purple for haptophytes. (TIF) [file pone.0086889.s004.tif]

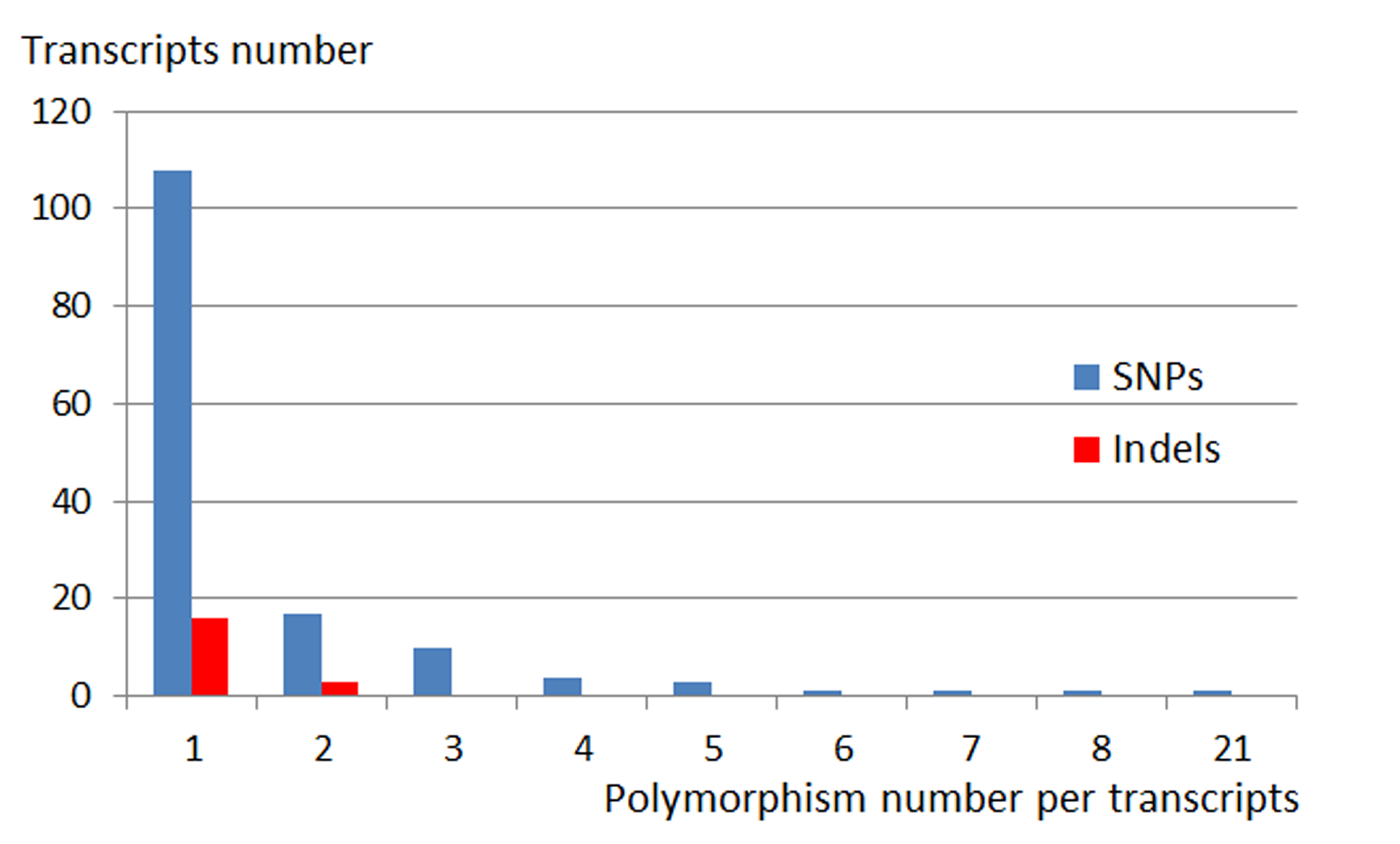

Supplement: Figure S4 — Distribution of positional polymorphisms (SNPs and indels) between Tiso-Wt and Tiso-S2M2 per transcripts. (TIF) [file pone.0086889.s005.tif]

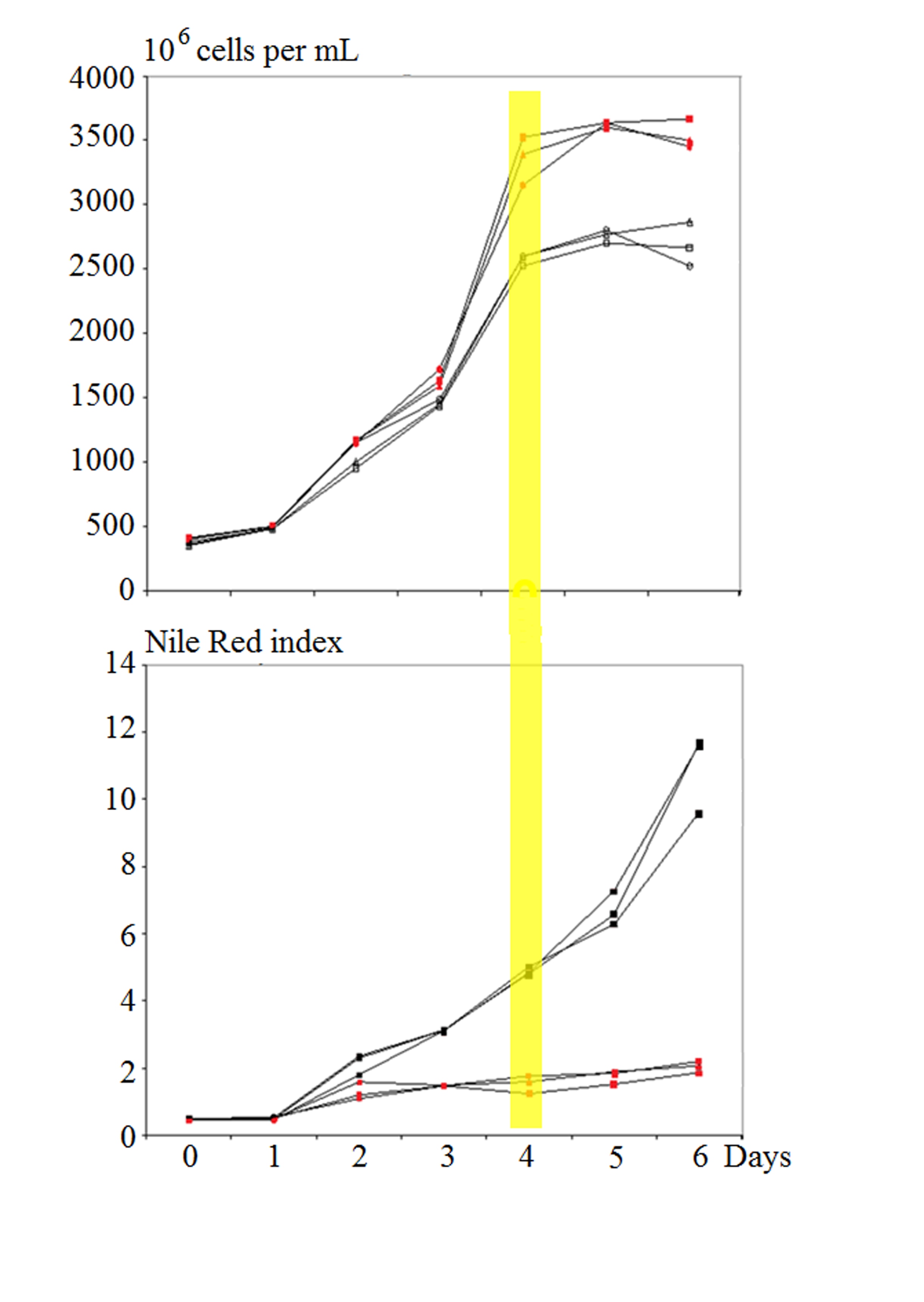

Supplement: Figure S5 — Growth of Tiso strains and lipid accumulation. Cells were counted with a Malassez counting cell and image analysis (SAMBA software). Lipid accumulation was estimated by measurement of Nile Red fluorescence by spectrofluorimetry as described by Bougaran et al., [22]. Red color for Tiso-Wt and black color for Tiso-S2M2. The yellow color shows sampling events. (TIF) [file pone.0086889.s006.tif]
